# Living with Stigma: Depressed Elderly Persons' Experiences of Physical Health Problems

**DOI:** 10.1155/2014/527920

**Published:** 2014-06-11

**Authors:** Anne Lise Holm, Anne Lyberg, Elisabeth Severinsson

**Affiliations:** Centre for Women's, Family and Child Health, Faculty of Health Sciences, Buskerud and Vestfold University College, P.O. Box 235, 3603 Kongsberg, Norway

## Abstract

The aim of this paper is to deepen the understanding of depressed elderly persons' lived experiences of physical health problems. Individual in-depth interviews were conducted with 15 depressed elderly persons who suffer from physical health problems. A hermeneutic analysis was performed, yielding one main theme, living with stigma, and three themes: longing to be taken seriously, being uncertain about whether the pain is physical or mental, and a sense of living in a war zone. The second theme comprised two subthemes, feeling like a stranger and feeling dizzy, while the third had one subtheme: afraid of being helpless and dependent on others. Stigma deprives individuals of their dignity and reinforces destructive patterns of isolation and hopelessness. Nurses should provide information in a sensitive way and try to avoid diagnostic overshadowing. Effective training programmes and procedures need to be developed with more focus on how to handle depressive ill health and physical problems in older people.

## 1. Introduction

Depression in older people has been reported to differ from that in younger people because of their preoccupation with physical health problems, which can lead to a risk that the depression will not be diagnosed and that the patient will not seek further help [1–4]. In addition, nurses and healthcare professionals frequently believe that depressed older persons' physical health problems are not real but a sign of depression. A possible consequence is that older persons with physical health problems and deprssion are misunderstood, discriminated against and abandoned by the healthcare system. Common pathways of depression and pain have been outlined in the literature [5]. Pain can give rise to physical incapacitation followed by depression due to reduced physical and social activities [5, 6]. Bonnewyn et al. [7] found that painful physical symptoms are associated with major depressive disorder and influence help seeking as well as use of medication. Suen and Tusaie [8] reported that depressed persons exhibited guilt, sadness, anger, resentment, loneliness, helplessness, hopelessness, inability to enjoy activities, and anhedonia. According to these authors [8], depressed elderly persons should be given time to express their emotional pain and distress, as they are capable of separating their mental state from their pain and physical health problems. Other studies have revealed that depressed older persons feel alienated from themselves and others, whose experiences pervade their whole life world, involving both body and soul [9]. Suffering from physical health problems has also been described as leading to an increased risk of suicide in older people [10, 11].

### 1.1. Background

Do depressed elderly persons experience stigma when it is believed that their physical health problems are not real but a symptom of depression? Stigma occurs when a person perceives that he/she is ignored and has been defined as a process whereby certain individuals and groups are unjustifiably rendered shameful, excluded, and discriminated against [12]. Stigmatising attitudes towards people with a range of mental disorders including depression have been investigated in studies undertaken in several countries [13]. One form of stigma is “diagnostic overshadowing” [14], defined as the misattribution of physical symptoms to a preexisting mental disorder [15]. Beliefs about and attitudes towards people with such disorders and the desire for social distancing have been explored [16, 17]. Experiences of discrimination (i.e., the behavioural aspects of stigma) have only been addressed in a few studies, until recently the focus was on serious mental illness, such as schizophrenia [18]. Lasalvia et al. [19] contributed to the understanding of depression-related discrimination by assessing the nature and severity of experienced and anticipated discrimination reported by over 1,000 persons with depression in 35 countries. However, Sirey et al. [20] found that although younger patients perceived more stigma than their older counterparts, it only predicted treatment discontinuation among the latter. A study by Roeloffs et al. [21] described stigma as being related to a number of chronic medical conditions, in addition to age, gender, ethnicity, and social support. The study by Evans-Lacko et al. [22] revealed that a national Time to Change programme against stigma and discrimination was successful in improving attitudes and behaviour but not knowledge among the general public in England. However, their study did not explore a possible relationship with age.

Stigma has been described as leading to the development of negative attitudes towards older persons such as prejudices, ageism, the creation of popular stereotypes and taboos, damaging self-beliefs, lack of public discussion, and alarmist popular and professional statements about burden and costs [23]. According to Sartorius [24], stigma leads to discrimination in the provision of services for physical health problems in older people with mental disorders. Thus, older people with a mental disorder carry a double burden, which merits special attention in order to prevent stigma and discrimination [12]. Even when municipal resources are available to support older persons who suffer from mental ill health, such individuals do not always seek care [23].

In 2012 and 2013, our research team conducted exploratory studies on data collected from in-depth [25, 26] interviews with depressed elderly persons from different parts of Norway. The result revealed that isolation and refusal to make use of available municipal resources mean that older persons with mental ill health suffer in silence [25, 26]. We explored depressed elderly persons' experiences of physical health problems because during the in-depth interviews the participants mentioned such problems in connection with depression.

### 1.2. Aim

To deepen the understanding of depressed elderly persons' lived experiences of physical health problems.

## 2. Methods

### 2.1. Design

The study has an interpretive hermeneutic approach [27] with in-depth interviews as the data collection method.

### 2.2. Participants

The participants comprised two men and thirteen women from the east and west coast of Norway. The inclusion criteria were persons diagnosed with a depressive or mood disorder, able to understand and speak the Norwegian language, resident in a community in Norway, referred to community healthcare during the previous six months, are over 60 years of age, and willing to speak about their experiences. Fourteen of the participants had been admitted to psychiatric hospitals where two were diagnosed with bipolar disorder. One participant was diagnosed as suffering from depression by a primary care physician. Their mean age was 69 years.

### 2.3. Data Collection

Data collection took place in community mental health centres in the east and west of Norway attended by patients with depression or a mood disorder. In 2011, a nurse manager in the community contacted the participants by telephone to arrange a date and time for an individual interview, which was held either in their own home or in the office of the first (ALH) or the second author (AL). The interview took the form of a dialogue between the interviewer and the participant where the interpreted meanings constituted the results. The authors encouraged the participants to narrate about their physical health problems and how they influenced everyday life. The opening question was, “Can you please describe your experiences of living with physical health problems?” 15 statements describing physical health problems were compiled in a table (Table 1). The interviews, which lasted between 60 and 120 minutes were audio-taped and transcribed verbatim.

### 2.4. Ethical Considerations

The Ethical Guidelines for Nursing Research in the Nordic Countries [28] were adhered to. Approval for the study was granted by The Regional Ethics Committee of Western Norway (no. 2010/2242). The interviews were conducted in a sensitive manner so as not to increase the depressed elderly persons' feelings of powerlessness and helplessness. They were provided with detailed written information andtheysigned a consent form and were assured that their name and identity would not be disclosed and that they had the right to withdraw at any time. All data were stored in a locked and fireproof filing cabinet.

### 2.5. Hermeneutic Analysis

Hermeneutic analysis means reflecting on one's understanding and the knowledge on which the analysis is based. It follows the hermeneutic principle of moving from the whole to the parts and back to the whole [27]. The hermeneutic circle was used to interpret the text. The authors reflected on the transcribed text and discussed divergent interpretations on several occasions before finally achieving consensus. The interview text was read again to ascertain whether a new meaning would emerge. Understanding the whole text can constitute a starting point for an analysis of its meaning in relation to a new aim or question. Sentences related to the aim were identified and grouped in a table alongside relevant abstractions, which formed the foundation of the search for themes representing the underlying meaning. The first author (ALH) reread the text to ensure that it had been correctly transcribed and that nothing was excluded. One main theme was identified, which represented our understanding of the abstracted content of the participants' statements. The hermeneutic circle is an essential tool for achieving new understanding and as researchers we experienced it as a back and forth movement between the whole and the parts of the interview text. Every sentence was related to the text as a whole in order to capture new meanings and expand the overall meaning (Table 2). The researchers reflected on, revised, discussed, and abstracted the text on several occasions, finally managing to identify a shared understanding that is intended to describe the participants' experiences.

### 2.6. Trustworthiness

As this study has a hermeneutic approach, it is important to establish trustworthiness, for which various strategies exist [29, 30]. The themes that emerged represent an attempt to understand the meaning of physical health problems and their influence on the depressed older persons' daily life. The epistemological notion was that the researchers and informants are interrelated and they interact with each other. The researchers influenced each other by discussing their reading and interpretation of the text on several occasions [29] as well as by being flexible and changing the theoretical perspective throughout the analysis, which is related to the concept of dependability [30]. We did not try to distance ourselves but attempted to treat the data in a neutral manner in accordance with the concept of confirmability. The term applicability as used by Öhman [30] relates to the concept of transferability and whether the knowledge gained in this study can be transferred to a similar context.

## 3. Results

One main theme, living with stigma, and three themes emerged: longing to be taken seriously, being uncertain about whether the pain is physical or mental, and a sense of living in a war zone. The second theme comprised two subthemes: feeling like a stranger and feeling dizzy. In the third theme, one subtheme emerged: afraid of being helpless and dependent on others.

### 3.1. Living with Stigma

Several of the participants described experiences where physical health problems and depression involved living with stigma, which can be understood as an experience of being considered different from others. This difference is caused by the discrimination these persons can experience when labelled with a mental illness such as depression, which frequently increases their sense of not being taken seriously or considered deviant. When depressed, older persons also suffer from physical health problems, which the literature describes as part of depression; healthcare providers seem to believe that the physical health problems are not real. A sense of being different can add to depressed elderly persons' suffering. Other people behave as if the elderly persons are imagining things and that their physical health problems are due to depression. Shame is often caused by living with stigma. However, the sense of shame can be hidden and not explicitly expressed. One of the participants stated;

*Because I suffer from major depression, my GP did not believe me when I developed a prolapse, despite the X-ray examination revealing a prolapse in my neck. He gave me medicine for depression. (Woman No. 1)*

Another participant reported that she went to two different GPs, but neither of them gave her a referral for an X-ray examination, as they did not believe that her pelvis was broken. She explained;

*Both doctors considered my health problems emotional or mental but not physical. So I finally had to call the ambulance myself. (Woman No. 4)*

#### 3.1.1. Longing to Be Taken Seriously

The participants were reluctant to mention depression, as they had the impression that it made healthcare professionals ignore their physical problems. They experienced being discriminated against and longed to be heard and believed. They revealed that GPs did not explain why their physical health problems were not examined. One of the participants was nearly blind and related that the healthcare professionals did not believe her when she complained about the lack of insulation in her council apartment, which made the floor cold. As she suffered from ischaemia, her feet gradually became blue and ice-cold. She stated;

*The nurses at the centre do not believe my story and want me to take anti-depressive medication. However, the GP uses such medication to calm the old people at the centre. I refuse to take it. And the last time the older persons were invited to a party, the nurse did not even look at me. (Woman No. 6)*

A female participant stated that she suffered from backache as a result of osteoporosis. She could not tolerate the pain medication, which she experienced as ineffective. She was diagnosed with the condition the previous summer, which was a relief as it explained her pain. Another of the participants described how she finally found a medical student who believed her and examined her for over one and a half hours. She stated;

*I was prescribed a medicine I was to take four times a day for 10 days. Nothing more. This helped me a lot. (Woman No. 13) *

#### 3.1.2. Being Uncertain about Whether the Pain Is Physical or Mental

Most of the participants reported uncertainty about whether their experiences of pain were physical or mental. Every day they woke up hoping that their health would improve. However, they found it impossible to plan everyday life, as the struggle between physical and emotional pain made their situation unpredictable, often exacerbated by lack of understanding on the part of the GP, other healthcare professionals, and their family members. One of the women had a pacemaker and also suffered from thyroid hormone problems, leading to fainting and hyperactivity. She related that she often argues with her GP about her situation and stated;

*I have read a lot about the pacemaker, and the thyroid hormone problem means that I'm sometimes hyper, which my GP relates to depression. I have to use a laxative every day, which makes me nervous because I never know when I will have to run to a toilet. It's awful, I wonder if all this is really related to my depression and anxiety? (Woman No. 14)*

Uncertainty due to the absence of examinations was explained as feeling that something is physically wrong despite being assured by the GP that everything is fine.

#### 3.1.3. Feeling Like a Stranger

Some of the participants explained that their physical pain made it almost impossible to fall asleep. At times they felt alienated from everyday life, characterized by the impression of being on the outside and not taking part in life. A combination of physical and emotional pain overwhelmed their body. One of the men reported that even when he was admitted to a psychiatric hospital, he did not tell anyone about his pain. He stated;

*You cannot go on and the emotional pain increases. How can I explain this to another person when I do not understand it myself? My whole body is aching. (Man No. 3)*

#### 3.1.4. Feeling Dizzy

Some were admitted to hospital because of their physical health problems. One woman who had been admitted to hospital twice because of dizziness explained;

*The GP told me they could not find anything wrong with me. But this was of no comfort because I'm still dizzy and my body continues to shake. I wonder is it tinnitus or is the dizziness related to my mental condition? (Woman No. 5)*

Another woman revealed that she could not turn her head in any direction without pain. She stated;

*I cannot clean my house because of the dizziness and pain in my head. I think this could be related to my brain tumour. I become so frightened about it that I'm hardly able to think. (Woman No. 13)*

#### 3.1.5. A Sense of Living in a War Zone

Most of the participants revealed that they live with a sense of impending disaster similar to being in a war zone and some thought that this was related to their depression. They were often extremely sensitive about the information they received from healthcare professionals about their physical conditions. One participant narrated that the medical specialist at the hospital told her that the cancer might spread to her brain, thus increasing the risk of death.

*After receiving this information I felt that I was in a war zone. It happened l8 years ago in January and I developed terrible anxiety. This information was a shock. The specialist asked me if there was anything I wanted to do before it was too late. I often think that had I not been informed about this, my life would have been different. (Woman No. 13)*

Many of the participants stated that they were terrified and had a feeling of disaster when informed that they were seriously ill. Catastrophic thoughts and feelings increased and one participant stated;

*One day I noticed a change in the skin above my lips. And I was right. The GP told me I had skin cancer that could easily spread to my brain and eyes. I became terrified because he told me that I could become blind. I developed an incredible anxiety that has followed me ever since. (Woman No. 4)*

#### 3.1.6. Afraid of Being Helpless and Dependent on Others

Many of the participants were able to manage life on their own. However, they were concerned that if they developed yet another illness it would increase their helplessness and make them dependent on others, which frightened them. One of the men stated;

*I have taken care of myself all my life, but after this stroke I have become more and more disabled, I cannot move and have no strength in my hand. I'm afraid of having another stroke that would make me even more helpless and dependent on others. (Man No. 11)*

A female participant explained that after an ankle fracture she was sent home in a state of psychosis because nobody understood how helpless it made her.

*I need an operation on my hips as well but they sent me home. However, my arms are weak and I found it almost impossible to climb the stairs. I just sit in my flat as there is nobody to help me. (Woman No. 9)*

The participants were exhausted due to worrying about their children or grandchildren and sometimes did not even know what they were worrying about. The worry was described as a constant feeling of restlessness. One of them stated;

*I'm so exhausted worrying about my sons and I'm constantly worried about everything. (Woman No. 8)*

## 4. Discussion

According to Goffman [31, page 3], stigma is “an attribute that is deeply discrediting.” Stigma is due to a perception of deviating from the social norm and involves a relationship between an attribute and a stereotype [31]. It leads to social sanctions whereby one person is labelled deviant, while another is considered normal and acceptable. The sanctions act as a social control mechanism by defining what is acceptable within a particular context. Stigma is an expression of people's responses to individuals who possess some undesirable characteristics. It can be discrediting and involve prejudicial and discriminatory practices [32, 33]. In several studies stigma has been found to reduce social functioning [34, 35]. Nurses and healthcare professionals can decrease stigma by taking depressed elderly persons' physical health problems seriously, thus alleviating their emotional pain and anxiety. For this reason, Jacoby [36] described stigma as a relational process. The experience of stigma can involve a sense of being an outsider, where connecting with others is challenging for older persons in their struggle to make sense of their despair [37]. Depressed elderly persons' experience of stigmatization may be a negative effect of the labelling process. The consequences of stigma for a person's life can be as harmful as the direct effects of the disease and serve as a barrier to recovery [38]. Being treated as a person who cannot be trusted can increase the feeling of inferiority and shame. Thomé et al. [33] demonstrated a correlation between stigma and poor functioning in bipolar disorder. Perceived stigma played a significant role in how the participants experienced their illness. A major difficulty in overcoming stigma, and probably one of its causes, is that it is next to impossible for those who have not experienced depression to understand the extent of the depressed elderly person's suffering. The emotional pain of depression is unimaginable to those who have not experienced it. Wolpert [39, page 223] stated that it may be too difficult to understand and that depressed individuals are seen as unpredictable persons who, “if they really tried, could pull themselves together.” It is difficult to believe what such unpredictable persons say about their physical health problems. Research has revealed that support from others can serve as a buffer against the development of depression and facilitate recovery, thus preventing isolation and withdrawal [38, 39]. Peer support groups have been described as one way of obtaining mutual respect [40].

*Longing to be taken seriously* can be related to the discrimination that is often experienced by depressed older persons. Stereotypes of depression might increase the negative attitudes of healthcare professionals. Depressed elderly people have to struggle to have their physical health problems believed. The phenomenon of “diagnostic overshadowing” [15, 41] refers to the fact that high levels of physical and mental ill health in elderly persons increase the risk of* not being taken seriously*. They are considered different and their physical health problems as imaginary and related to depression. Such stigma can be associated with being discriminated against and judged [34]. Lack of sensitivity in the provision of information can also increase the stigma of depression. When one is made to feel that one deviates from the norm, one becomes ashamed. Perceived stigma involves internal shame associated with being depressed as well as fear of stigma; one feels unworthy. Sometimes being in a depressed state can imply a lack of self-management, a sense of estrangement, and loss of togetherness [25]. In this state, depressed persons have no strength to fight for their rights. Dignity is described as a special dimension of value. One is worthy of respect from others as well as from oneself [42]. Barker [43] stated that self-determination is the most significant human right. It is an existential dimension of being in the world related to experiences of freedom and dignity. In even the most difficult situations it is possible to increase a depressed elderly person's strength by enhancing her/his self-determination and dignity.

*Being uncertain about whether the pain is physical or mental* seemed to involve an experience of insecurity, which was exacerbated by not knowing what the day would bring in terms of physical and emotional pain. The participants became increasingly uncertain of their own experiences because who can one trust when one cannot trust oneself? Uncertainty has been described in the literature as a feeling related to insecurity and distrust. The review by Hansen et al. [44] provided a synthesis of patient experiences of uncertainty in illness. The included studies revealed a sense of unpredictability when living with physical health problems such as chronic hepatitis, HIV and AIDS, endometriosis, and stroke [44]. None of the studies included depression. However, Hansen et al. [44] suggested that nurses and healthcare professionals need to promote the experience of control, confidence, and insight among patients. It seems that the older persons' struggle increased their confusion, emotional pain, and distress. Such painful conditions require a deeper understanding of and insight into the complex interrelated issues of soma and psyche. Research has demonstrated that pain and depression are related to many socioeconomic factors [5]. Chou and Chi [45] found that pain was an important predictor of depression in elderly Chinese primary care patients. Another study revealed that older persons who experience pain are at risk of developing depression and vice versa [46]. If one is exposed to such suffering, one can feel like a stranger, even in a familiar environment. When perceptions of physical health problems are not confirmed, an individual can become estranged from her/himself and unsure of her/his own feelings and experiences. Are they real? Am I really experiencing this? Who can confirm it? Do I have pain or are my physical health problems a figment of my imagination?

It appears that complaints of* dizziness* are often misunderstood by mental health professionals in primary care, who seem to perceive it as a sign of depression. However, this may be relevant, as research has revealed that a large percentage of patients presenting with dizziness develop secondary psychiatric disorders over the course of their disease. In particular, clinical depression and anxiety are health problems in older persons [47]. Is dizziness related to depression? Sczepanek et al. [48] stated that little is known about the course of incident dizziness.

*A sense of living in a war zone* can be related to anxiety and disaster associated with physical health problems, especially serious diseases such as cancer. Anxiety and depression in the elderly are often related to the burden of serious illness [49]. Depression is often associated with past loss and negative thoughts about the self and the world, while anxiety is linked to future harm and danger [49]. Research has revealed that depressed older persons are capable of differentiating emotional pain and distress from physical pain [8]. Anxiety can be related to the fear and worry of* being increasingly dependent on others *and is understandable in the context of older persons' existential situation. It raises the question about how long one will be able to maintain one's autonomy and independence. Worrying can be related to depression, anxiety, and thoughts about future disasters. Depressed older persons seem to have fewer mental and social resources to distract them from catastrophic feelings about the future. Research has demonstrated that they increasingly worry about many things and that the type and degree of worry vary across the life span [50]. More intense worries are related to greater severity of depression among the oldest old. Emotional support from adult children had a positive effect on depression and worries, while social support was important for understanding one's catastrophic feelings about the future [50, 51]. Human beings have a desire to make sense of their life—as opposed to merely solving problems. Such a desire can change the focus from ill health and illness to the human being, giving a person time and space to talk about her/his previously unarticulated worries related to grief and sadness. Personal growth can continue into old age [52]. However, older persons may be more sensitive about being taken seriously, and it does not take very much to weaken an individual's potential and optimism. The existential dimension of being in the world need not be related to complete freedom from worries.

## 5. Recommendations

### 5.1. Nursing Practice

This study revealed that depressed elderly persons who suffer from physical health problems are vulnerable as they seem to have a double burden due to experiencing negative attitudes, discrimination, and stigma. Nursing practice must provide information in a sensitive way and always ascertain whether physical health problems are real as opposed to a sign of depression. Due to the cost of clinical interventions, depressed older persons pose a difficulty as they constitute a group with many physical health problems. It is necessary to develop an action plan to address their physical health problems because physical diseases increase in old age.

### 5.2. Nursing Research

This study highlighted the fact that discrimination means any distinction, exclusion, or preference that has the effect of nullifying or impairing equal enjoyment of rights [12]. Thus such individuals must be taken seriously in order to lessen the burden of stigma [4]. According to Martinez-Arán et al. [35], the label “depressed” or “depressive” has consequences such as sanctions associated with fear and lack of understanding on the part of society. The problem of “diagnostic overshadowing” must be investigated and educational interventions developed to target it. It is important to establish a consensus among general practitioners, nurses, and health care professionals about how to address these issues. De Mendonça Lima [23] suggested that coordinated efforts are necessary to reduce stigma and discrimination among older persons who suffer from mental disorders. Now is the time to develop more coordinated efforts and to employ a range of research approaches. Nurses and healthcare professionals need to address prejudices and negative attitudes towards depressed older persons, especially if having the impression that the person in question is trying to explain her/his struggle by means of physical health problems.

## 6. Conclusion

Reducing stigma has been on the agenda for at least ten years in most countries. Stigma deprives individuals of their dignity and reinforces destructive patterns of isolation and hopelessness. A greater understanding and acceptance of depressed older persons with physical health problems is necessary. Nurses and healthcare professionals need more education about how negative self-beliefs held by those who are or might be stigmatised can increase shame, low self-esteem, and unwillingness to discuss problems or access services. Effective training programmes and procedures should be developed with greater focus on how to handle depressive ill health and physical problems in older people. More research is required on nonstigmatising treatment and care for depressed older persons with physical health problems and pain.

## Figures and Tables

**Table 1:** Physical health problems reported by the participants.

Participants	Age	Physical health problems	Gender
1	61	Neck pain, prolapse, infection in the optic nerve	Female
2	64	Stomach ache	Female
3	67	Back pain, poor vision, diabetes mellitus, heart attack, kidney stone	Man
4	73	Hypertension, arthritis, spondylitis, skin cancer, impaired hearing	Female
5	65	Tinnitus (ortopediese)	Female
6	92	Hip pain, poor blood circulation, impaired hearing	Female
7	74	Osteoporosis and pain when walking	Female
8	69	Hypertension, obesity, pain related to colon trouble (obstipation)	Female
9	62	Pain following ankle fracture	Female
10	82	Arthrosis of the knees and hips, diabetes mellitus, hypertension	Female
11	64	Apoplexia cerebri, cataract/poor vision	Man
12	62	Cataract/poor vision	Female
13	63	Low metabolism, brain tumour, osteoporosis	Female
14	69	Thyroid hormone problems	Female
15	72	Back pain, osteoporosis	Female

**Table 2:** Living with stigma: depressed elderly persons' experiences of physical health problems and depression.

Main theme	Living with stigma		
Themes	Longing to be taken seriously	Being uncertain aboutwhether the pain is physical or mental	A sense of living in a war zone

*Subthemes *		Feeling like a stranger	Afraid of being helpless and dependent on others

		Feeling dizzy	
